# Bioinformatics analysis and development of functional markers for *TaMYB4-1A* in wheat

**DOI:** 10.1371/journal.pone.0319980

**Published:** 2025-04-15

**Authors:** Dan Zhao, Yuru Ma, Yufeng Yang, Zhaoyang Li, Chaoran Wang, Yuhan Fu, Yang Chen, Tengteng Zhang, Yi Ding, Huiqiang Wang, Xuehui Zhang, Hao Zhang

**Affiliations:** 1 College of Life Sciences, Hengshui University, Hengshui, China; 2 School of Chemical Engineering and Biotechnology, Xingtai University, Xingtai, China; 3 Ministry of Education Key Laboratory of Molecular and Cellular Biology, Hebei Research Center of the Basic Discipline of Cell Biology, Hebei Collaboration Innovation Center for Cell Signaling and Environmental Adaptation, Hebei Key Laboratory of Molecular and Cellular Biology, College of Life Sciences, Hebei Normal University, Shijiazhuang, China; 4 Seed Management Station of Handan City, Handan, China; Government College University Faisalabad, PAKISTAN

## Abstract

MYB transcription factors play crucial roles in various stages of plant growth and development. Bioinformatics analysis revealed that wheat TaMYB4-1A contains two conserved MYB domain. The coding region of TaMYB4-1A is 792 bp, encoding 263 amino acids. TaMYB4-1A is a hydrophilic protein, and its encoded protein is localized in the cell nucleus. Evolutionary tree analysis indicates that the TaMYB4 protein shares the closest relationship with Aegilops, barley, and rye. Tissue-specific expression analysis revealed that *TaMYB4-1A* is expressed in wheat roots, stems, leaves, and seeds 14 days post-flowering, with the highest expression in the seeds. Promoter cis-acting element analysis showed that the promoter region of *TaMYB4-1A* contains various cis-acting elements, including meristem regulatory elements, drought-induced elements, and hormone response elements. qRT-PCR analysis showed that the expression of *TaMYB4-1A* is suppressed under high salinity and PEG treatment, suggesting that *TaMYB4-1A* may play a critical regulatory role in response to salt and drought stress. There are two haplotypes of *TaMYB4-1A*, namely *Hap-1A-I* and *Hap-1A-II*. The average plant height of varieties with haplotype *Hap-1A-I* is significantly higher than that of varieties with haplotype *Hap-1A-II*. This research provides a basis for future in-depth investigation of the biological function of the *TaMYB4-1A* gene and offers promising candidate genes for molecular marker-assisted wheat breeding.

## Introduction

Wheat (*Triticum aestivum* L.) is one of the most vital food crops in China and worldwide, ranking second globally in production after rice and maize, accounting for approximately 35% of the world’s total food production [1], and providing the largest source of protein in human diets. High temperatures and drought caused by global warming, along with the excessive use of fertilizers, have resulted in a 50% decrease in wheat yield [2–6]. The MYB transcription factors represent one of the largest protein families in plants [7,8]. The N-terminal DNA-binding domain (DBD) repeat sequences (Rs) of MYB transcription factors exhibit significant conservation. These repeats are composed of around 52 amino acid residues, which fold into three α-helices. The helix-turn-helix (HTH) structure is formed by the second and third helices. The C-terminal region is more variable than the N-terminal and is involved in regulatory functions [9]. Multiple studies have shown that MYB proteins play a role in regulating abiotic stress tolerance within plant genomes. *MYB* genes such as *OsMYB2*, *OsMYB3R-2*, *OsMYB4*, and *OsMYBS3* in rice and *TaMYB2*, *TaMYB32*, *TaMYB56*, *TaMYB30*, and *TaMYB73* in wheat have been identified as key components of plant abiotic stress response pathways [10–13]. In plants, the hormone ABA plays a pivotal role in regulating responses to drought stress, facilitating adjustments in plants under water scarcity through a complex genetic regulatory network. To mediate stress adaptation, ABA-dependent signaling pathways have been shown to function through the involvement of MYC and MYB transcription factors [14–18]. MYB transcription factors positively regulate plant growth under salt stress by enhancing antioxidant defenses and regulating the formation of the leaf cuticle [19]. High temperatures also constrain crop growth, and MYB transcription factors can enhance transgenic plant heat tolerance by upregulating genes associated with amino acid metabolism, which boosts amino acid metabolism [20]. Drought and high salinity are abiotic stress factors that cause severe economic losses in agriculture [21]. Soil salinization restricts plant growth and development, and plants adapt to salt stress through various regulatory mechanisms, including salt-responsive signaling pathways, plant hormone signal transduction, generation of reactive oxygen species (ROS), and mechanisms for maintaining ion homeostasis. ZmKTF1 regulates the DNA methylation level in the promoter region of genes and affects gene expression under salt stress [22]. Drought and salt stress induced the expression level of *IbMYB330* [23]. Therefore, exploring MYB family genes is crucial for enhancing wheat stress tolerance and achieving high and stable yields. This study predicts the function of the *TaMYB4-1A* gene by analyzing its sequence and protein structure and examines its tissue-specific expression in wheat. The expression of this gene under high salinity and drought conditions was clarified, laying a foundation for further investigation of the molecular mechanisms by which *TaMYB4-1A* responds to environmental stress. By detecting the polymorphisms of the *TaMYB4-1A* sequence, molecular markers were developed, and excellent haplotypes were identified through associations with agronomic traits, providing superior candidate genes for marker-assisted wheat breeding.

## 1 Materials and methods

### 1.1 Experimental materials

The wheat material used in this study was Kenong 9204 (KN9204). The material used for gene polymorphism detection and agronomic trait association analysis consists of a natural population comprising 323 wheat varieties, which are mainly distributed in the Northern Winter Wheat Region and the Huang-Huai Winter Wheat Region of China. The natural population was planted under 10 different environmental conditions (year × location × environment) at the Shunyi and Changping in 2015 and 2016, Five plants were randomly selected from each material to measure phenotypic data such as spike length, number of spikelets per spike, number of grains per spike, thousand-grain weight, yield per plant, and plant height.

RNA extraction reagent RNAiso Plus (9108) was purchased from Takara.

The RNA reverse transcription kit (11141ES60) and premixed solution for real-time quantitative PCR amplification Hieff® qPCR SYBR Green Master Mix (Low Rox Plus) (11202ES08) were purchased from Yeasen Biotechnology (Shanghai) Co., Ltd.

Hoagland’s modified basal salts (H353) were purchased from PhytoTech.

Agarose (1110GR100) was purchased from Biofroxx.

### 1.2 Experimental methods

#### 1.2.1 Bioinformatics analysis and primers used.

*TaMYB4-1A* gene information was retrieved using Ensembl Plants; cis-regulatory elements of *TaMYB4-1A* were analyzed using PlantCARE; protein physicochemical properties were analyzed using Expasy; secondary structure of TaMYB4-1A protein was analyzed using SOPMA; tertiary structure of TaMYB4-1A protein was analyzed using SWISS-MODEL; protein hydrophobicity was analyzed using ProtScale; phosphorylation sites in the TaMYB4-1A amino acid sequence were predicted using NetPhos-3.1; subcellular localization of TaMYB4-1A was predicted using CellPLoc2.0; a phylogenetic tree of TaMYB4 protein was constructed using MEGA7.0 software; real-time quantitative PCR primers were designed using Primer5. Agronomic trait association analysis was performed using TASSEL5 software. The primers used in this experiment (Table 1) were synthesized by Sangon Biotech (Shanghai) Co., Ltd.

**Table 1:** Primers used in this study.

Primer name	Primer sequence (5’-3’)
qRT-TaMYB4-1A-F	AGCTCGGAGGCGGTCACTGAC
qRT-TaMYB4-1A-R	AAGTCTATTATGTCTCCGCC
TaGADPH-F	TTAGACTTGCGAAGCCAGCA
TaGADPH-R	AAATGCCCTTGAGGTTTCCC
TaMYB4-1A-F1	TCTTGGGGTCGGTGATTACCGCCGATTACT
TaMYB4-1A-R1	CTTTTGTCTTCTTTGGAGTATTGCTCCGAT
TaMYB4-1A-dCAPs-F	CCGCCTCCATCAGACCCTCGGCACC
TaMYB4-1A-dCAPs-R	ACGCTGGCAAGACAGTTAAATGTTT

#### 1.2.2 Expression analysis of the *TaMYB4-1A.*

Select full and uniform KN9204 wheat seeds, soak them in 1% sodium hypochlorite for 10 minutes, rinse three times with distilled water, place them on culture dishes with moist vermiculite, and vernalize them at 4°C for 4 weeks. Then plant them in pots, and collect roots, stems, leaves, and seeds on the 14th day after flowering for gene expression pattern analysis.

Select plump and uniform KN9204 wheat seeds, soak in 1% sodium hypochlorite for 10 minutes, rinse three times with distilled water, and place them on culture plates with two layers of filter paper in an artificial climate chamber. The culture conditions are 22°C light for 16 h and 18°C dark for 8 h. After 3–4 days of culture, transfer the seedlings to liquid Hoagland medium. Once the seedlings reach the one-leaf stage, subject them to different stress treatments: 200 mM NaCl and 20% PEG6000 to simulate drought stress. Collect samples at 0, 1, 3, 6, 9, 12, and 24 hours to detect *TaMYB4-1A* gene expression under stress. Store the samples at -80°C for later use.

#### 1.2.3 Real-time quantitative PCR.

Total RNA from wheat root was extracted using the Trizol method with RNAiso Plus reagent (9108, Takara); reverse transcription into cDNA was performed using the RNA reverse transcription kit (11141ES60, Yeasen). Real-time quantitative PCR was performed using Hieff® qPCR SYBR Green Master Mix (Low Rox Plus) (11202ES08, Yeasen). Specific real-time quantitative PCR primers qRT-TaMYB4-1A-F/qRT-TaMYB4-1A-R (Table 1) were designed based on the T*aMYB4-1A* gene sequence, and wheat *TaGAPDH* was used as the internal reference gene to calculate the relative expression level of *TaMYB4-1A*. The reaction system contained 1 μL of cDNA, 0.5 μL of each forward and reverse primer, 10 μL of Hieff® qPCR SYBR Green Master Mix (Low Rox Plus), and 8 μL of ddH_2_O, with a total volume of 20 μL. The reaction program was as follows: pre-denaturation at 95°C for 5 min; denaturation at 95°C for 10 s, annealing at 60°C for 30 s, for 40 cycles; the melt curve followed the default settings of the IBM7500 instrument. Three biological replicates were set, and the relative expression level of the target gene under different stress conditions was calculated using the 2^-^△△^Ct^ method.

#### 1.2.4 Marker development and genotyping of *TaMYB4-1A.*

The polymorphism of the *TaMYB4-1A* sequence was analyzed using the Wheat Genome Variation Database, and cluster analysis identified two haplotypes of *TaMYB4-1A*. Based on the SNP site analysis, the *Eco*RII restriction enzyme was developed as a dCAPs molecular marker targeting the SNP at position 353 (C/A), which can differentiate the two haplotypes of *TaMYB4-1A*. After two rounds of PCR, *Eco*RII was used for enzyme digestion, and 4% agarose gel electrophoresis was performed for detection.

#### 1.2.5 Association analysis.

Using molecular markers, the natural population was scanned, and the phenotypic data of agronomic traits over multiple years and locations were analyzed for association with genotyping results using the TASSEL5 software.

## 2 Results and analysis

### 2.1 Sequence analysis of *TaMYB4-1A* in wheat

**Fig 1:**
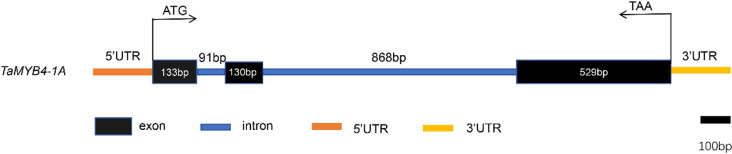
Analysis of *TaMYB4-1A* gene sequence.

The *TaMYB4-1A* gene sequence was analyzed using Ensembl Plants, revealing a total length of 1751 bp, consisting of 3 exons and 2 introns. The lengths of the 3 exons are 133 bp, 130 bp, and 529 bp, respectively. The coding region covers 792 bp, with a non-coding region of 959 bp, encoding 263 amino acids in total (Fig 1).

### 2.2 Physicochemical properties and hydrophilicity/hydrophobicity analysis of TaMYB4-1A

**Fig 2:**
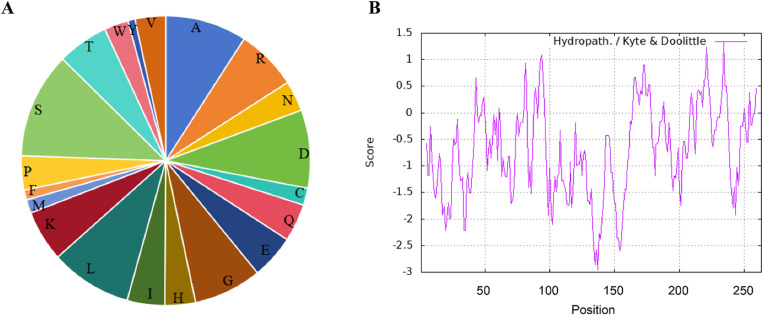
Amino acid composition and the hydrophilicity/hydrophobicity analysis of TaMYB4-1A. (A) Amino acid composition of TaMYB4-1A. A: alanine; R: arginine; N: asparagine; D: aspartic acid; C: cysteine; Q: glutamine; E: glutamic acid; G:glycine; H: histidine; I: isoleucine; L: leucine; K: lysine; M: methionine; F: phenylalanine; P: proline; S: serine; T: threonine; W: tryptophan; Y:tyrosine; V: valine.; (B) The hydrophilicity/hydrophobicity analysis of TaMYB4-1A. Score>0, indicates hydrophobicity; Score < 0, indicates hydrophilicity.

The physicochemical properties of the TaMYB4-1A protein were predicted using ProParam. Results showed that the protein consists of 20 amino acids, with serine having the highest content at 31 residues, accounting for 11.8% of the total amino acids, followed by alanine and leucine, each with 24 residues, making up 9.1% of the total (Fig 2A). The molecular formula of the protein is C_1227_H_1961_N_377_O_404_S_9_, with 3978 atoms, a relative molecular weight of 28,746.89, and a theoretical isoelectric point (pI) of 6.31. The number of positively charged amino acid residues (Arg+Lys) is 33, while the number of negatively charged residues (Asp+Glu) is 36. The hydrophilicity/hydrophobicity of TaMYB4-1A was analyzed using ExPASy-ProtScale, revealing an uneven distribution of hydrophilic and hydrophobic amino acids across the sequence, with an average hydrophilicity index of -0.693, indicating it is a hydrophilic protein (Fig 2B).

### 2.3 Protein Structure Analysis of TaMYB4-1A

**Fig 3:**
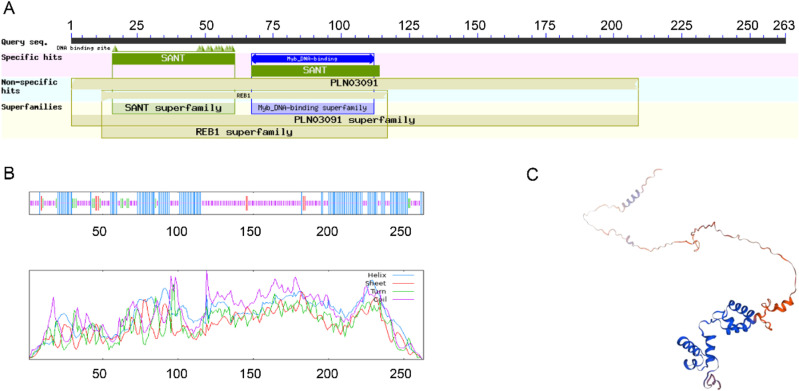
Analysis of TaMYB4-1A protein structure. (A)TaMYB4-1A protein domain; (B)TaMYB4-1A protein secondary structure, Bule: Helix; Red: Sheet; Green: Turn; Purple: Coil; (C) TaMYB4-1A protein tertiary structure.

The domain analysis of TaMYB4-1A protein via NCBI shows that there are highly conserved MYB (SNAT) domains at amino acid positions 16–61 and 67–112, indicating that TaMYB4-1A is a typical R2R3-type MYB transcription factor (Fig 3A). Secondary structure analysis of the TaMYB4-1A protein using SOPMA showed that the protein is primarily composed of alpha helices, beta turns, extended strands, and random coils. The alpha helices consist of 103 amino acids (39.16%), beta turns consist of 16 amino acids (6.08%), extended strands consist of 8 amino acids (3.04%), and random coils consist of 136 amino acids (51.71%) (Fig 3B). Alpha helices are the most stable among the secondary structures of proteins, and the higher the proportion of alpha helices, the more stable the protein. Using the Phyre2 online tool to predict the tertiary structure of TaMYB4-1A protein, the results indicated that random coils are the predominant component, followed by alpha helices (Fig 3C).

### 2.4 Prediction of phosphorylation sites and subcellular localization of TaMYB4-1A

**Fig 4:**
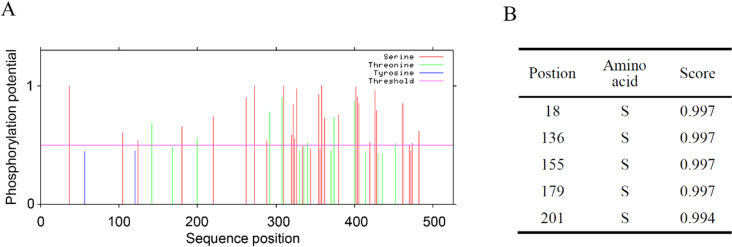
Prediction of TaMYB4-1A protein phosphorylation sites. (A) phosphorylation site prediction of proteins. (B) the amino acid sites with phosphorylated predictive values higher than 0.99.

Phosphorylation site prediction of TaMYB4-1A amino acids using NetPhos-3.1 showed 58 phosphorylation sites with scores above the threshold value of 0.5, distributed unevenly, including 26 serine sites, 9 threonine sites, and no tyrosine sites (Fig 4A). The predicted phosphorylation scores for serine residues at positions 18, 136, 155, 179, and 201 were all above 0.990, much higher than the threshold of 0.5 (Fig 4B). Subcellular localization prediction of TaMYB4-1A using Cell-PLoc2.0 showed that the protein is localized to the nucleus, where it functions.

### 2.5 Signal peptide prediction of TaMYB4-1A protein

**Fig 5:**
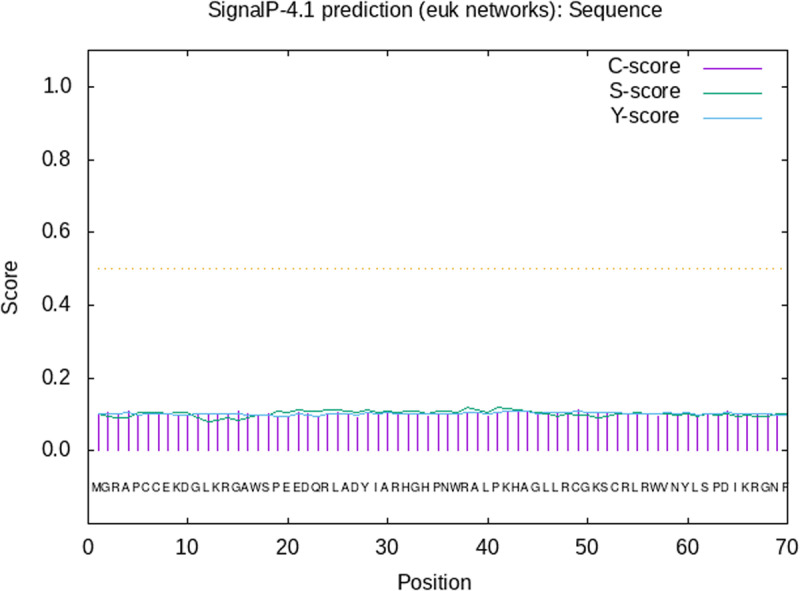
Prediction of signal peptide of TaMYB4-1A protein.

A signal peptide is a special sequence located at the N-terminus of a secretory protein, which guides the newly synthesized protein into the endoplasmic reticulum or Golgi apparatus, ensuring that the protein is transported to the correct organelle or secreted outside the cell. The presence of a signal peptide is an important characteristic of secretory proteins. The signal peptide prediction for the protein encoded by the TaMYB4-1A gene using NetPhos-4.1 indicated that the protein lacks a signal peptide (Fig 5), suggesting that the TaMYB4-1A encoded protein is a non-secretory protein.

### 2.6 TaMYB4 phylogenetic tree construction

**Fig 6:**
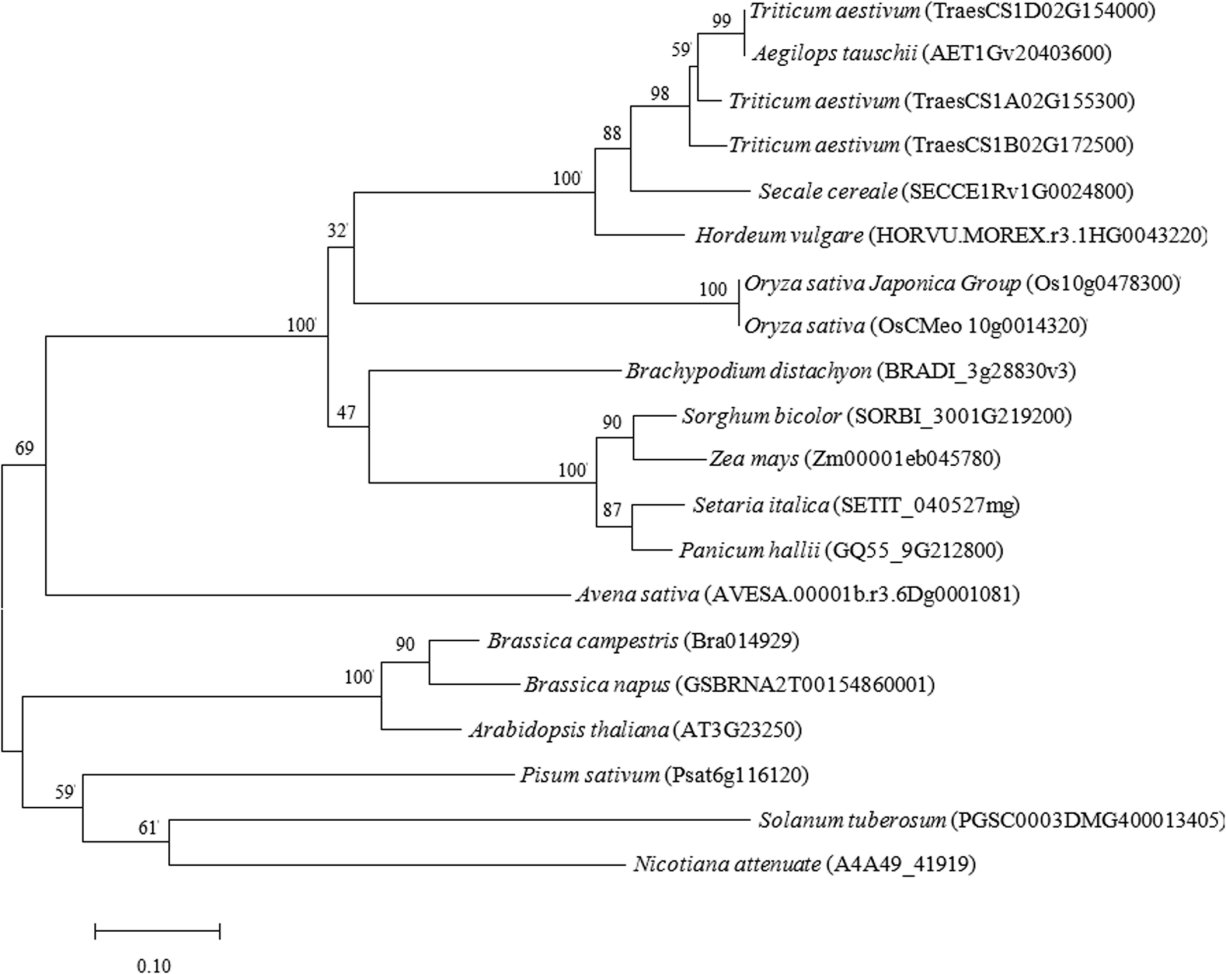
Phylogenetic tree of TaMYB4. The phylogenetic tree was generated using MEGA 7 software based on amino acid sequence comparison.

The amino acid sequence of wheat TaMYB4 was compared with other species’ sequences from the Ensembl Plants database to identify similar sequences, and a phylogenetic tree was constructed. The results (Fig 6) indicate that the 20 species formed two major clusters, with the TaMYB4 protein having higher similarity to proteins from *Aegilops tauschii*, *Hordeum vulgare*, and *Secale cereale*, indicating closer evolutionary relationships, while showing lower similarity to *Avena sativa* and *Nicotiana attenuata*.

### 2.7 Cis-acting element analysis of the *TaMYB4-1A* promoter

Using PlantCare to analyze the cis-acting elements in the promoter region of *TaMYB4-1A*, the results indicate that the promoter region of this gene may have certain impacts on plant growth, stress responses, and exogenous hormone processes (Table 2).

**Table 2:** Putative cis-acting regulatory elements in the promoter region of *TaMYB4-1A.*

Cis-acting regulatory element	Frequency	Biological function
ABRE	3	ABA response element
ARE	5	cis-acting regulatory element essential for the anaerobic induction
Box 4	3	part of a conserved DNA module involved in light responsiveness
CAAT-box	38	common cis-acting element in promoter and enhancer regions
CAT-box	1	cis-acting regulatory element related to meristem expression
CGTCA-motif	5	cis-acting regulatory element involved in the MeJA-responsiveness
G-Box	2	cis-acting regulatory element involved in light responsiveness
GATA-motif	1	part of a light responsive element
A-box	2	cis-acting regulatory element
CCAAT-box	1	MYBHv1 binding site
MYB	4	MYB binding site
MBS	1	MYB binding site involved in drought-inducibility
TCA-element	1	cis-acting element involved in salicylic acid responsiveness
TATA-box	29	core promoter element around -30 of transcription start
TGACG-motif	5	cis-acting regulatory element involved in the MeJA-responsiveness

In addition to core promoter elements like TATA-box, A-box, and CAAT-box, the promoter also contains important anaerobic induction elements (ARE), MYB protein-binding elements (MYB), CCAAT-box for MYBHV1 protein binding, drought-induced element MBS, and other stress-response related elements. It also includes light-responsive elements (Box4, G-Box, GATA-motif), meristem regulation element (CAT-box), abscisic acid response element (ABRE), salicylic acid response element (TCA-element), and hormone response elements like the methyl jasmonate response elements TGACG-motif and CGTCA-motif.

This suggests that *TaMYB4-1A* may play an important role in wheat growth, stress responses, and hormone signaling.

### 2.8 Tissue-specific expression analysis of *TaMYB4-1A* gene

**Fig 7:**
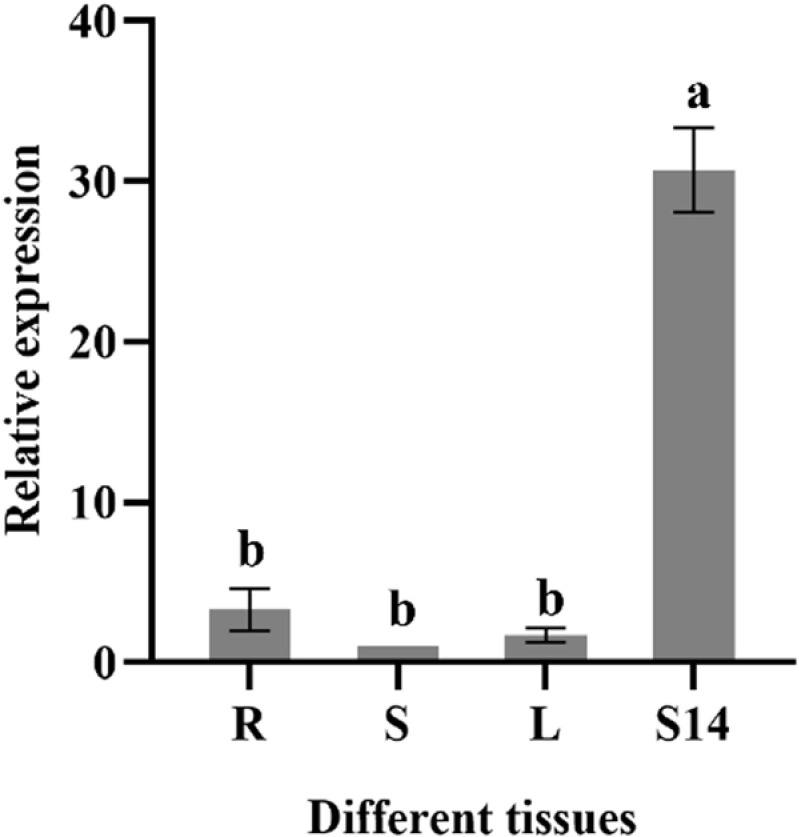
The relative expression of *TaMYB4-1A* gene in different tissues. Note: R:root, S:stem, L:leaf, S14: Seeds at 14 days after flowering. Data represent mean ± S.D. of three biological repeats. The data analyzed by one-way ANOVA with Tukey’s significant difference test, statistically significant differences are indicated by different lowercase letters (P<0.05).

To determine the expression of *TaMYB4-1A* in different wheat tissues, qRT-PCR analysis was performed. *TaMYB4-1A* was expressed in wheat roots, stems, leaves, and seeds at 14 days post-flowering, with the highest expression observed in seeds at 14 days post-flowering (Fig 7). This indicates that *TaMYB4-1A* plays an important biological role in wheat seed development.

### 2.9 Expression analysis of *TaMYB4-1A* under stress conditions

**Fig 8:**
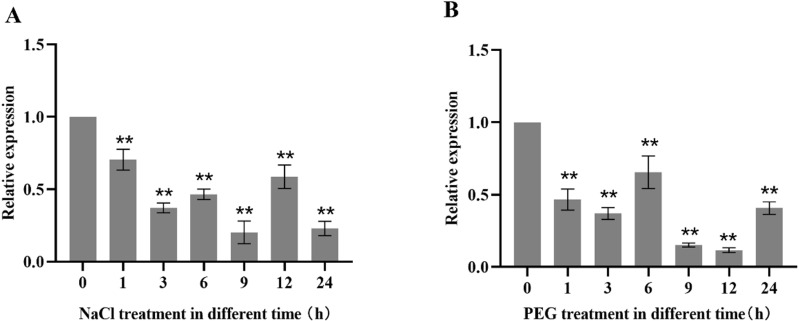
The expression of *TaMYB4-1A* under salt and drought treatments in wheat. (A)The expression of *TaMYB4-1A* under 20% PEG. (B) The expression of *TaMYB4-1A* under 200 mM NaCl. Data represent mean ± S.D. of three biological repeats. The data analyzed by one-way ANOVA with Tukey’s significant difference test. *P < 0.05, **P < 0.01.

To determine whether *TaMYB4-1A* is involved in the response to stress conditions, the gene expression was analyzed in wheat seedlings under salt and drought treatments. The results showed that under 200 mM NaCl treatment, *TaMYB4-1A* expression was significantly downregulated, reaching its lowest level at 9 hours(Fig 8A). Under 20% PEG treatment, *TaMYB4-1A* expression was significantly downregulated, with the lowest expression at 12 hours (Fig 8B). These results indicate that *TaMYB4-1A* is inhibited by salt and drought stress.

### 2.10 Development of molecular markers for *TaMYB4-1A* and genotyping

**Fig 9:**
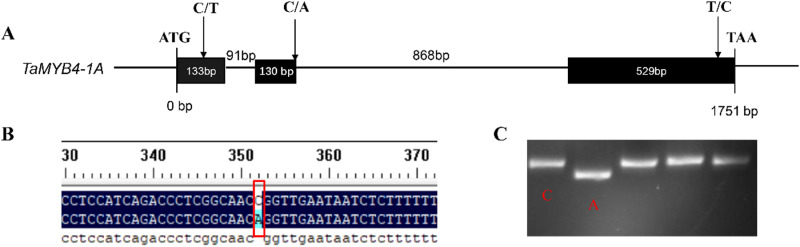
Molecular marker development of *TaMYB4-1A.* (A) The schematic diagram and polymorphism sites of *TaMYB4-1A*. (B) Molecular marker development of *TaMYB4-1A*, the red box represent two haplotype base differences. Red box indicates base differences between two haplotypes. (C) two haplotype PCR products were digested by *Eco*RII.

By performing polymorphism analysis on the exon sequence of *TaMYB4-1A* using the Wheat Union Database, three SNP sites were identified in the exon region of *TaMYB4-1A*: 73 (C/T), 353 (C/A), and 1672 (T/C). These SNP sites divide *TaMYB4-1A* into two haplotypes: *Hap-1A-I* and *Hap-1A-II* (Table 3, Fig 9A,B). Using the dCAPs Finder 2.0 website, a molecular marker was designed for the SNP located at the 353rd exon position (C/A) by introducing a mismatch at one base, creating an *Eco*RII restriction site. After two rounds of PCR, the *Eco*RII enzyme was used for digestion. If the 353rd position is C, the site cannot be cut by the enzyme, whereas if it is A, the site can be cut by the enzyme (Fig 9C).

**Table 3:** The polymorphism of *TaMYB4-1A.*

No.	Site	Variation type	*Hap-1A-I*	*Hap-1A-II*
1	73	SNP	C	T
2	353	SNP	C	A
3	1672	SNP	T	C

### 2.11 Association analysis of *TaMYB4-1A* haplotypes with agronomic traits

The natural population was planted in 10 different environments at two wheat experimental bases in Shunyi and Changping during 2015 and 2016, resulting in phenotypic data on thousand-grain weight, single plant yield, plant height, number of spikes per plant, grains per spike, and grain weight.

**Fig 10:**
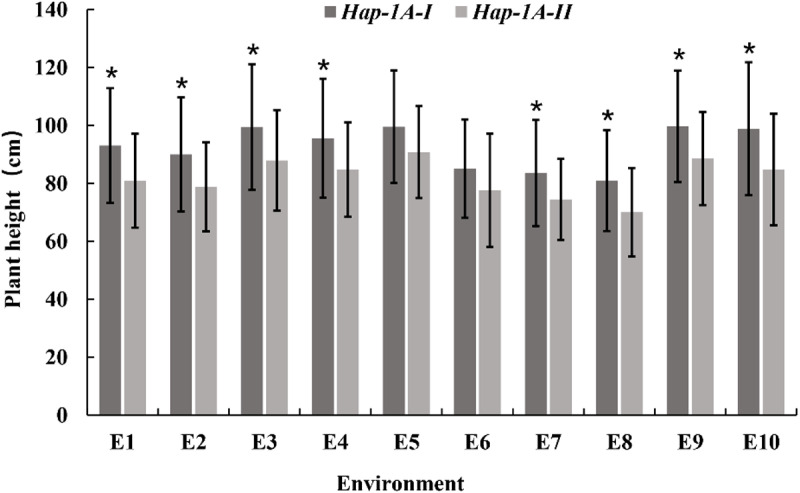
Plant height of cultivars with different *TaMYB4-1A* haplotypes in ten growing environments. Data represent mean ± S.D. of three biological repeats. The data analyzed by one-way ANOVA with Tukey’s significant difference test. *P<0.05.

Among the two haplotypes of *TaMYB4-1A*, *Hap-1A-I* and *Hap-1A-II* accounted for 94% and 6%, respectively, in the 323 samples of the natural population. Through association analysis of agronomic traits in the natural population, the results showed significant differences (P < 0.05) in plant height between the two haplotypes of *TaMYB4-1A* under 8 environmental conditions (Table 4). The average plant height of varieties with the *Hap-1A-I* haplotype was significantly higher than that of varieties with the *Hap-1A-II* haplotype. As shown in Table 4, the plant height variations caused by polymorphic site mutations were minimally affected by environmental conditions. This suggests that different haplotypes of *TaMYB4-1A* are associated with plant height (Fig 10).

**Table 4:** Association analysis of *TaMYB4-1A* with Plant height.

Years	No.	Location	Condition	Plant heigh *P*-value
2016	E1	Shunyi	Drought stress and heat stress	0.01*
	E2	Shunyi	Drought stress	0.012*
	E3	Shunyi	Well-watered and heat stress	0.02*
	E4	Shunyi	Well-watered	0.02*
	E5	Changping	Well-watered	0.062
	E6	Changping	Drought stress	0.157
	E7	Shunyi	Drought stress and heat stress	0.022*
2015	E8	Shunyi	Well-watered and heat stress	0.013*
	E9	Shunyi	Drought stress	0.016*
	E10	Shunyi	Well-watered	0.012*

## 3 Discussion

MYB transcription factors are found in all eukaryotes and can be divided into four categories based on the number of MYB domain repeats: 1R-MYB, R2R3-MYB, 3R-MYB, and 4R-MYB. MYB transcription factors form one of the largest protein families in plants [24–26]. So far, 204 MYB transcription factors have been identified in Arabidopsis thaliana and 244 in rice [27]. Research has shown that the MYB transcription factor family plays a crucial role in plant responses to stress. *AtMYB41*, *AtMYB74*, *AtMYB102*, and *AtMYB108* are key factors in drought and salt tolerance in Arabidopsis thaliana [28]. The wheat gene *TaMYB-CC5* shows specific expression in roots and is significantly upregulated under phosphorus deficiency and drought stress. Overexpression of *TaMYB-CC5A* in Arabidopsis leads to a significant increase in root length under stress conditions, enhancing tolerance to phosphorus deficiency and drought [29]. The R2R3-type MYB transcription factor *MYB110* in rice, induced by low phosphorus stress, does not affect phosphorus absorption and accumulation, but its mutation leads to a significant increase in plant height and reduces the plant height response to low phosphorus stress [30]. In this study, promoter cis-acting element analysis revealed that the promoter region of *TaMYB4-1A* contains response elements for plant hormones such as abscisic acid, salicylic acid, and methyl jasmonate; drought-induced elements; and meristem-related elements. Tissue expression patterns and the expression of *TaMYB4-1A* under stress treatments indicate that this gene has the highest expression in seeds 14 days after flowering and is involved in responses to drought and salt stress.

Wheat is one of the most important staple crops for global food security, primarily grown in arid and semi-arid regions, where it is highly susceptible to water shortages. Studies have shown that a 40% reduction in water usage can lead to a 20.6% decrease in wheat yield, and factors like global warming, salinity, and other abiotic stresses, along with continued population growth, will further exacerbate wheat yield reductions [4,31–33]. Therefore, in wheat breeding, developing new varieties that are salt-alkali-tolerant, drought-resistant, heat-tolerant, and exhibit stable high yields under stress conditions is a key objective. With the significant innovations in genomic technology and the development of molecular biology tools, the discovery of superior allelic variations and the development of functional markers can accelerate the selection and breeding of new wheat varieties, improving breeding efficiency [34].

With the sequencing of various plant genomes, genome-wide association studies (GWAS) have shown that genetic variations in both coding and non-coding regions can lead to phenotypic differences in traits [35,36]. Through candidate gene association analysis, it was found that a 26 bp insertion or deletion (InDel574) in the coding region of the wheat gene *TaDTG6-B* is significantly associated with drought resistance variability during the seedling stage of wheat. Compared to TaDTG6-B^In574^, TaDTG6-B^Del574^ exhibits stronger transcriptional activation, enabling its encoded DREB protein to have higher protein interaction capacity and to bind with DRE/CRT cis-elements, thereby regulating the expression of downstream genes. Silencing the TaDTG6-B^Del574^ gene reduces the drought resistance of transgenic wheat, while its overexpression significantly enhances the drought resistance of transgenic wheat. Expression changes in the TaDTG6-B^In574^ allele do not affect drought resistance [37]. In this study, three SNP sites were detected in the coding region of *TaMYB4-1A*, which divide *TaMYB4-1A* into two haplotypes: *Hap-1A-I* and *Hap-1A-II*. Agronomic trait association analysis showed that the different haplotypes of *TaMYB4-1A* are associated with plant height. Plant height is a key trait that affects crop yield [38]. Further research is needed to explore the relationship between plant height and traits such as yield and lodging resistance.
